# From Stress to Strength: Fostering a Positive Learning Climate to Promote Learning, Performance, and Well-Being

**DOI:** 10.5811/westjem.63706

**Published:** 2026-08-21

**Authors:** Juhi Varshney, Esther Chen

**Affiliations:** * Emory University, Department of Emergency Medicine, Atlanta, Georgia; † University of California San Francisco, Department of Emergency Medicine, San Francisco, California

## INTRODUCTION

The emergency department (ED) can be a challenging context for learners and teachers. Emergency physicians face high rates of burnout and attrition, which impact our ability to teach and learn on shift.^1^,^2^ Anxiety, fear, and uncertainty have been associated with increased diagnostic error among clinicians and impede learning in trainees.^3^,^4^

In contrast, fostering a positive learning climate offers a number of potential benefits. The broaden-and-build theory posits that when we experience positive emotion, we broaden our mental awareness and we build our capacity for creativity, resilience, and social connection.^5^ Tapping into emotion on shift can help residents reflect on new learning goals, and enjoying our shifts can improve patient care.^6^–^8^ For trainees in the ED, positive emotion may also help promote psychological safety, which has been associated with increased patient safety and reduced burnout.^9^–^10^ Positive emotion promotes good health; healthcare professionals who experience awe regularly report less stress and increased well-being.^11^ Finally, after a post-pandemic decline in medical students applying to emergency medicine (EM), applicants who chose EM were more likely to have had a positive, early clinical experience in the ED.^12^

The challenges of caring for patients in a complex clinical environment demand systemic solutions, but individual educators have the power to shape the learning experience more than we may realize. Learners notice our enthusiasm for teaching and patient care, reporting that it is one of the hallmarks of an exceptional teacher.^13^ The psychology literature presents evidence-based strategies that can promote a positive learning climate on shift to enhance trainee learning, performance, and well-being. Every shift has space to explore and model small practices that help us share the joy of EM.

## 10 TIPS

Consider the following 10 strategies to promote a positive learning climate in the ED:

1. **Connect interpersonally with learners:** Relationships are the foundation of social support and belonging. Many physicians report that these relationships are integral to their happiness at work.^14^ Social support from attending supervisors has been shown to help residents recover from difficult cases and medical errors.^15^–^17^ Even during a brief moment of down time between patients, we can ask questions about our learners, and we can share some details about ourselves to reciprocate. Building trust with trainees promotes psychological safety and motivates them to learn.
2. **Draw on emotional contagion**. The ED is a place of heightened emotions, and we absorb the fear, frustration, and grief around us. But the converse is true too. Positive emotions like awe, gratitude, and contentment are also contagious.^18^ When we truly enjoy our work, we spread that emotional state with our team, our learners, and our patients. Consider your emotional state on shift and identify moments when you can share your positive emotions with others. Pair your learner with colleagues who display enthusiasm on shift to spread positive energy within the team.
3. **Cultivate flow:** Flow is deep immersion in an activity, the joy of finding rhythm in a task and letting the world fall away.^19^ Early studies on flow found that people experience flow more while working rather than in leisure.^20^ The clinical environment presents opportunities for flow on every shift, but flow needs the proper balance of challenge and ability. If the challenge exceeds the learner’s ability, they experience anxiety instead of flow. As teachers, we can identify challenges that are appropriate for each learner’s level, help learners define their goals, and then coach them when they need to troubleshoot. While we cannot force flow to happen, we can cultivate conditions that welcome it.
4. **Engage with play and humor:** The high stakes of our work are not necessarily at odds with having fun. Play emerges in how we relate to our work, and a playful approach finds ways to make the task at hand more enjoyable. Creating space for play can boost productivity.^21^ Appropriate use of humor with teaching can enhance affect, attention, and learning.^22^ In the clinical setting, humor can reduce stress in trainees and contribute to camaraderie.^23^ This approach must be exercised with caution as negative humor can disengage learners. Create inside jokes with your learners, develop fun traditions for each shift, be willing to laugh at yourself—and you might boost the learning process.
5. **Savor the sacred moments:** Our work is filled with awe. Sacred moments are marked by great meaning, spiritual dimensions, and feelings of peace. Examples include deep listening, comforting an especially vulnerable patient, or guiding a family through end-of-life care. Sacred moments can arise unexpectedly on shift, and noticing sacred moments is associated with lower burnout.^24^ We can savor these by taking a few moments to pause, breathe, and be present. Savoring practices may improve well-being and decrease distress.^25^ Some researchers have used this practice to nurture creativity and engagement for their learners.^26^ On shift, we can name sacred moments when they occur and encourage our learners to savor them before moving on to their next tasks.
6. **Express your appreciation:** When we appreciate our learners, they experience improved confidence, job satisfaction, and professional identity, all of which help them deliver better patient care.^27^ Many programs have formal initiatives to recognize residents’ outstanding clinical cases.^28^ Individual attendings can promote this practice. Most shifts hold opportunities to appreciate the hard work of our trainees. Taking an extra moment to articulate what we genuinely value about our learners can support their learning and well-being. Sharing good saves at resident conference and department meetings can foster a culture of appreciation at the program level as well.
7. **Stimulate the senses**. Our senses are a portal to relief during stressful moments on shift. Music therapy has been linked to emotional regulation and stress relief in the workplace.^29^ Touch interventions have been shown to reduce cortisol levels.^30^ On shift, this may look like putting on a warm jacket or placing a hand on the heart.^31^ Aromatherapy and the taste of comfort foods are also associated with stress relief.^32^,^33^ Pausing to smell a cup of coffee before taking a sip, eating a favorite snack, or playing fun music can improve our mood, making us more present for patient care and teaching. We can role model these practices for learners and invite them to reconnect with their senses too.
8. **Take a break:** Short breaks help us reset while also boosting performance.^34^,^35^ Taking a break can improve attention and performance on challenging tasks, without hurting efficiency or patient safety.^36^,^37^ Sunlight exposure correlates with improved mental health for healthcare workers; therefore, a microbreak to step outside provides both cognitive and physiological benefits.^38^ Taking a break is a learned skill but one we can practice ourselves and model for our learners.^39^
9. **Learn from positive deviance**. Positive deviance is an asset-based approach that recognizes creative, sometimes unexpected approaches to difficult circumstances.^40^ Positive deviance invites us to notice what others do well and to use positive outliers as a roadmap for our own future behavior. Learning through positive deviance has been shown to improve clinical performance of medical students and well-being of residents.^41^,^42^ On shift, we can highlight successful cases that we may take for granted with the same attention that we pay to cases that did not turn out so well. Case-based error review is a standard part of the residency curriculum, but we can also examine cases with good outcomes using a similar rigor to learn from positive deviance as well.
10. **Practice self-compassion**. Medical training can encourage self-criticism and harsh self-talk. Sometimes this feels like the only way we can improve. Self-compassion offers an alternate approach to personal development.^43^,^44^ Self-compassion has three dimensions: self-kindness; shared humanity; and mindfulness.^44^ Teachers who practice self-compassion have been found to support their learners’ autonomy more than teachers who do not practice self-compassion.^45^ When we show ourselves the compassion that we give to our patients and our learners, we create more internal space to care and teach.

## DISCUSSION AND LIMITATIONS

*“I always believed that students should enjoy learning” bell hooks* 46

Every shift in the ED is different. Competing clinical responsibilities and frequent interruptions can detract from the space and energy needed for the strategies mentioned above. But promoting a positive learning climate does not need to be grand or time-consuming; instead, clinicians can make small choices that turn toward joy and connection when opportunities arise. Rather than attempting all these strategies at once, we recommend implementing one practice that might work well with your clinical environment and your learner on each shift. While every shift may not be good, there is good in every shift. Educators must also be realistic; while they can cultivate a positive learning climate, learners ultimately control their own emotional state.

We recognize that the clinical learning environment is subject to forces beyond the individual educator’s control. Many of the challenges that learners and educators face on shift require systemic solutions beyond these strategies. The clinical learning environment is shaped by the institution, and creating truly positive learning climates requires significant support from the hospital system and may even require a departmental or institutional culture shift.

While the positive-psychology literature suggests strategies that may be easily implemented into our ED workflow, limited empirical evidence exists regarding their effectiveness in clinical practice. Future research should examine how these practices can promote learning, well-being, and performance for emergency physicians.

## CONCLUSION

Cultivating a positive learning climate on shift in the ED can foster learning, enhance performance, and nurture well-being for both educators and learners. We describe multiple strategies from the positive- psychology literature that clinical educators can add to their educational repertoires. Naming these practices encourages broader dissemination of their use, greater experimentation, and future studies of their impact. Emotion is an important component of medical education. Collating these 10 strategies is a starting point to consider how we can all promote a positive learning climate.

## Figures and Tables

**Table:** Ten tips to promote a positive learning climate in the emergency department.

Strategy:	Why this strategy works:	How this strategy might look in practice:
Connect interpersonally with learners	Social support builds trust, promotes psychological safety, and motivates learners.	Ask residents about their lives or interests outside of medicine and share a few personal details of your own.
Draw on emotional contagion	Positive emotions like enthusiasm, gratitude, and awe can easily spread to those around us.	Consider your emotional state on shift and identify moments when you can share your enthusiasm or gratitude with your team.
Cultivate flow	The flow state is deep immersion in an activity where one loses track of time. For learners to experience flow, the challenge must match their ability.	Encourage learners to identify their learning goals, assign them to patients that are appropriate for their skill level, and coach them as challenges arise.
Engage with play and humor	A playful approach makes tasks enjoyable, enhances attention, and boosts productivity.	Create inside jokes with your learners, develop fun traditions for each shift, and be willing to laugh at yourself.
Savor the sacred moments	Reflecting on and sharing sacred moments, such as providing comfort to a patient or guiding a family through end-of-life care, can foster feelings of peace.	Identify and share sacred moments as they occur to help trainees recognize them. Savor them before moving on to the next task.
Express your appreciation	When learners feel appreciated, they report improved confidence, job satisfaction, and professional identity–which all enhance patient care.	Articulate moments that you truly appreciate your learner during a shift. Share “good saves” with program leadership and at resident conference.
Stimulate the senses	Engaging different senses promotes stress relief during shift.	Pause to smell the coffee before taking a sip, eat a favorite snack, or play a favorite tune during shift—and encourage your learners to do the same.
Take a break	Taking a short break can improve attention, boost performance, and improve mental health. This is not intuitive for most learners, and we must model it for them.	Step outside for a few moments to get fresh air and sunlight. Encourage your learner to take a break during shift as well.
Learn from positive deviance	Noticing what others do well can improve our clinical performance.	Highlight and review successful cases with the same attention that you pay to cases that did not turn out so well.
Practice self-compassion	Self-compassionate teachers promote learner autonomy. Self-compassion creates more internal space to care and learn.	During difficult moments on shift, model self-kindness, mindfulness, or shared humanity instead of self-criticism.
